# Bufei Yishen granule combined with acupoint sticking improves pulmonary function and morphormetry in chronic obstructive pulmonary disease rats

**DOI:** 10.1186/s12906-015-0787-0

**Published:** 2015-08-08

**Authors:** Yange Tian, Ya Li, Jiansheng Li, Yang Xie, Minghang Wang, Yuqiong Dong, Linlin Li, Jing Mao, Lili Wang, Shan Luo

**Affiliations:** Institute for Geriatrics, Henan University of Traditional Chinese Medicine, Zhengzhou, 450046 Henan Province China; The collaborative innovation center for Respiratory Diseases Diagnostics, Treatment and New Drug Research and Development, Zhengzhou, 450046 Henan Province China; Central Laboratory, the First Affiliated Hospital, Henan University of Traditional Chinese Medicine, Zhengzhou, 450008 Henan Province China; Institute for Respiratory Diseases, the First Affiliated Hospital, Henan University of Traditional Chinese Medicine, Longzihu University Town, Zhengdong New District, Zhengzhou, 450008 Henan Province China

**Keywords:** Chronic obstructive pulmonary disease, Bufei Yishen granule, Acupoint sticking, Morphology, Traditional Chinese medicine

## Abstract

**Background:**

The integrated therapy of Bufei Yishen granule and acupoint sticking has been used in the treatment of stable chronic obstructive pulmonary disease (COPD) clinically, with remarkable benefits. This study was initiated to observe the effects of the combination of Bufei Yishen granule and acupoint sticking on pulmonary function and morphormetry in a COPD rat model.

**Methods:**

Rats were randomized into Control, Model, Bufei Yishen (BY), Acupoint sticking (AS), Bufei Yishen + Acupoint sticking (BY + AS) and aminophyline (APL) groups. COPD rats were duplicated by repeated cigarette smoke and bacterial exposures. The rats were treated with normal saline, Bufei Yishen granule, acupoint sticking, Bufei Yishen + Acupoint sticking and aminophylline, respectively, from week 9 through 20. Pulmonary function was measured by using a whole body plethysmograph every 4 weeks. The rats were sacrificed at the end of week 20, and lung tissue histology and ultrastructure was observed under light and electron microscopes.

**Results:**

The pulmonary function, including tidal volume (V_T_), peak expiratory flow (PEF) and expiratory flow at 50 % tidal volume (EF50), was markedly decreased from week 8 in COPD rats (*P* < 0.05). At week 20, V_T_, PEF and EF50 were significantly lower in Model group (*P* < 0.05). Compared with Model group, V_T_, PEF and EF50 were higher in BY and BY + AS groups (*P* < 0.05), and EF50 was higher in AS group, while V_T_ was higher in APL group (*P* < 0.05). Markedly histological and ultrastructural changes, including respiratory membrane thickening, volume density of lamellar corpuscle decreasing, mitochondria reducing in type II alveolar cell, were found in COPD rats and were alleviated in the treated groups, especially in BY and BY + AS groups.

**Conclusion:**

Bufei Yishen granule and acupoint sticking can improve pulmonary function and lung pathological impairment in COPD rats, the curative effect of the combination is better than acupoint sticking or aminophylline only.

## Background

Chronic obstructive pulmonary disease (COPD), a common preventable and treatable disease, is characterized by persistent airflow limitation that is usually progressive and associated with an enhanced chronic inflammatory response in the airways and the lung to noxious particles or gases [1]. It has been a major serious disease threatening public health because of its increasing incidence, mortality and heavy economic burdens. At present, various treatment methods, such as low-dose and slow-release theophylline, inhaled β2 agonists and corticosteroids, as well as health education, pulmonary rehabilitation, have been used in clinical treatment. However, it is difficult to keep the symptoms from progression without suffering lots of side effects or adverse events [2]. In recent years, more and more evidences showed that Traditional Chinese Medicine (TCM), including internal and external therapies, has potential advantages in improving symptoms, reducing the frequency of acute exacerbation, improving quality of life in stable COPD [3, 4]. Bufei Yishen granule, a special prescription for lung-kidney qi deficiency syndrome (a major syndrome in stable COPD patients), has been confirmed curative in the treatment of stable COPD, which can improve lung function and immunological function of patients with COPD [5]. Shu-Fei Tie, an ointment for acupoint sticking of external therapy improved from an effective prescription from ancient China, can excite vital qi in human body and is clinically used in the treatment of chronic pulmonary diseases, and is also proved effective in COPD prevention with its safety, convenience and fewer side effects [6]. In our previous study, Bufei Yishen granule combined with acupoint sticking therapy showed beneficial effects in reducing the frequency and duration of acute exacerbation, alleviating symptoms and improving quality of life in patients with stable COPD [7, 8]. In this study, we intended to observe the effect of the combination of Bufei Yishen granule and Shu-Fei Tie on pulmonary function and morphology in COPD rats, and provide a basis for further study to explore the mechanism of the integration of internal and external therapy.

## Methods

### Animals

Sixty male and 60 female Sprague Dawley rats, specific pathogen-free, weighing (200 ± 20) g, 2-month-old, were purchased from Laboratory Animal Center of Henan Province (SCXK [Henan] 2010–0002). Rats were housed in the individual ventilated cages (Fengshi, Suzhou, China) seven days before experiment, freely access to sterile food and water. Experimental protocols were approved by the Experimental Animal Care and Ethics Committees in the First Affiliated Hospital, Henan University of Traditional Chinese Medicine, Zhengzhou, China.

### Bacteria

*Klebsiella pneumoniae* (strain: 46114) purchased from National Center for Medical Culture Collection (Beijing, China), was cultured, harvested and prepared into normal saline solution, 6 × 10^8^ colony forming units (CFU) per milliliter (mL), before administrated to animals [9].

### Cigarette

Hongqiqu® filter cigarette, containing tar 10 mg, nicotine 1.0 mg and carbon monoxide 11 mg, was purchased from Henan Tobacco Industry Co., Ltd., (Zhengzhou, China).

### Drugs

Bufei Yishen granule (consisted of Ginseng Radix et Rhizoma 9 g, Astragali Radix 15 g, Corni Fructus 12 g, Epimedii Herba 9 g, Lycii Fructus12 g, Schisandrae Chinensis Fructus 9 g, etc.) were prepared and provided by the Department of Pharmacology in the First Affiliated Hospital, Henan University of Traditional Chinese Medicine, Zhengzhou, China.

Shu-Fei Tie (consisted of Semen Brassicae 10 g, Rhizoma Corydalis 5 g, Rhizoma Zingiberis 5 g, Asarum Heterotropoides 5 g, Daphne Genkwa 10 g, etc.), 3.0 g/tubes, were produced by the Department of Pharmacology in the First Affiliated Hospital, Henan University of Traditional Chinese Medicine, Zhengzhou, China.

Aminophylline Tablets (Xinhua, Shandong, China), 0.1 g/tablet, were crushed before administrated to animals.

### Grouping and COPD model preparation

Rats were randomized into Control, Model, Bufei Yishen (BY), Acupoint sticking (AS), Bufei Yishen + Acupoint sticking (BY + AS) and aminophyline (APL) groups. COPD rats were duplicated by cigarette smoke and bacterial exposures according to reference [9], and evaluated whether it was made successfully or not according to the symptoms, pulmonary function [10]. Rats were exposed to tobacco smoke of 8 cigarettes during the first two weeks, twice a day and 15 cigarettes from week 3 to 12, three times a day. *Klebsiella* pneumoniae solution (0.1 mL) was slowly dropped into the two nostrils in an alternate fashion, every 5 days in the first 8 weeks.

### Administrations

From week 9 through 20, the rats in Control and Model groups were intragastrically administrated with normal saline (2 mL/animal, b.i.d) and applied Shu-Fei Tie placebo (2 times/week); Bufei Yishen granule (4.44 g/kg/d, b.i.d) and Shu-Fei Tie placebo were administrated to BY group; normal saline and Shu-Fei Tie (2 times/week) was administrated to AS group; Bufei Yishen granule and Shu-Fei Tie was given to BY + AS group, while aminophyline (2.3 mg/kg•d, b.i.d) and Shu-Fei Tie placebo was used in APL group. Dosages adjustments were made every week according to body mass. The equivalent dosages were calculated by the formula: D_rat_ = D_human_ × (I_rat_/I_human_) × (W_human_/W_rat_)^2/3^. D: dose; I: body shape index; W: body weight. The rats in each group were sacrificed at week 20.

**Fig. 1:**
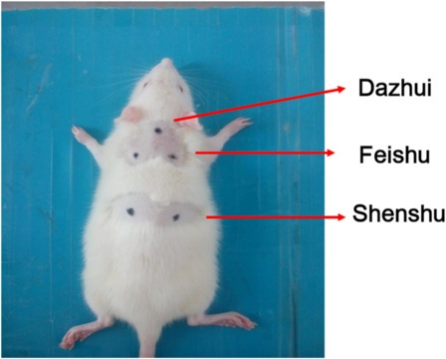
Dazhui, Feishu and Shenshu acupoint location on rat

The Shu-Fei Tie was applied on Dazhui (GV14), Feishu (BL13) (both sides), Shenshu (BL23) (both sides) (Seen in Fig. 1) [11]. GV14: the 14th point in Governor Vessel; BL13: the 13th point in bladder meridian; BL23: the 23rd point in bladder meridian. After rats were anesthetized mildly, the hair around the acupuncture points (1.5 cm × 1.5 cm) was shaved and then unhaired with Na_2_S (80 g/L) for 3 min. Shu-Fei Tie ointment (0.1 g/point) or Shu-Fei Tie placebo (0.1 g/point) was put and pressed gently onto the acupoints and covered with medical adhesive tapes, on each Monday and Thursday. Each treatment lasted for 4 ~ 6 h.

In case of the skin was burnt by Shu-Fei Tie ointment, the broken skin were disinfected with alcohol wipes gently and wrapped with gauze. If only one side of Feishu or Shenshu was burnt, the other side would be applied as usual. If both sides of Feishu or Shenshu were injured and not healed up, these acupoints would be stopped for one time. The rats would be excluded if one or more acupoints were stopped two or more times.

### General status

General status of rats was observed during the experiment, including activity, breath, fur, especially mouth and nasal secretion, breath conditions and food and water intake.

### Pulmonary function tests

Tidal volume (V_T_), peak expiratory flow (PEF) and expiratory flow at 50 % tidal volume (EF50) were measured by an unrestrained whole body plethysmograph (Buxco, NC, USA) at week 0, 4, 8, 12, 16, 20.

### Lung morphology

#### Pathological changes

After formalin lavaged and fixed for 72 h, the lung tissues was cut into 3-mm thick sections, and then embedded in paraffin and sliced into 4-μm slices. Hematoxylin-eosin (HE) stain was performed and photograph was captured by a PM-10 AD optical microscope (Olympus, Japan) and the pathological changes were observed and evaluated. The alveolar cavity and the density of alveoli were determined as follow. Mean linear intercept (MLI) (μm) = L/Ns. After a cross (+) was drawn through the center of each photo, the number of alveolar septum (Ns) laid on the cross was counted, and then the total length of the cross (L) was measured. Mean alveolar numbers (MAN) (/mm^2^) = Na/A. The number of pulmonary alveoli in each visual field (Na) and the area of the visual field (A) was measured [12].

#### Ultrastructure

After the 1 mm^3^ of lung tissues were successively fixed in 4 % glutaraldehyde and osmic acid, gradient dehydrated, transparentized in dimethylbenzene and embedded with Epon-812, ultrathin sections (50 nm) were prepared by ultramicrotome. Ultrastructural changes of respiratory membrane and type II alveolar epithelial cell were observed with JEM-1400 electron microscope (Olympus, Japan). Mitochondrial volume density (Vv), specific surface area (δ) and membrane area (δm), and volume density (Vv) of lamellar body were measured.

### Statistical analysis

SPSS 19.0 software (IBM; Armonk, NY, USA) was used for data analysis. Data are expressed as mean ± SEM. One-way analysis of variance (ANOVA) was employed for multiple comparisons. *P* < 0.05 was considered significantly statistical difference.

## Results

### General conditions

From the third week, the fur of COPD model rats was withered and became yellow. The rats gradually became weak and asthenia, and showed mucous hypersecretion, anorexia, body weight reduction, hydrouria, diarrhea. From week 12, the symptoms of rats in treatment groups were alleviated, especially in BY and BY + AS groups.

### Pulmonary function

**Fig. 2:**
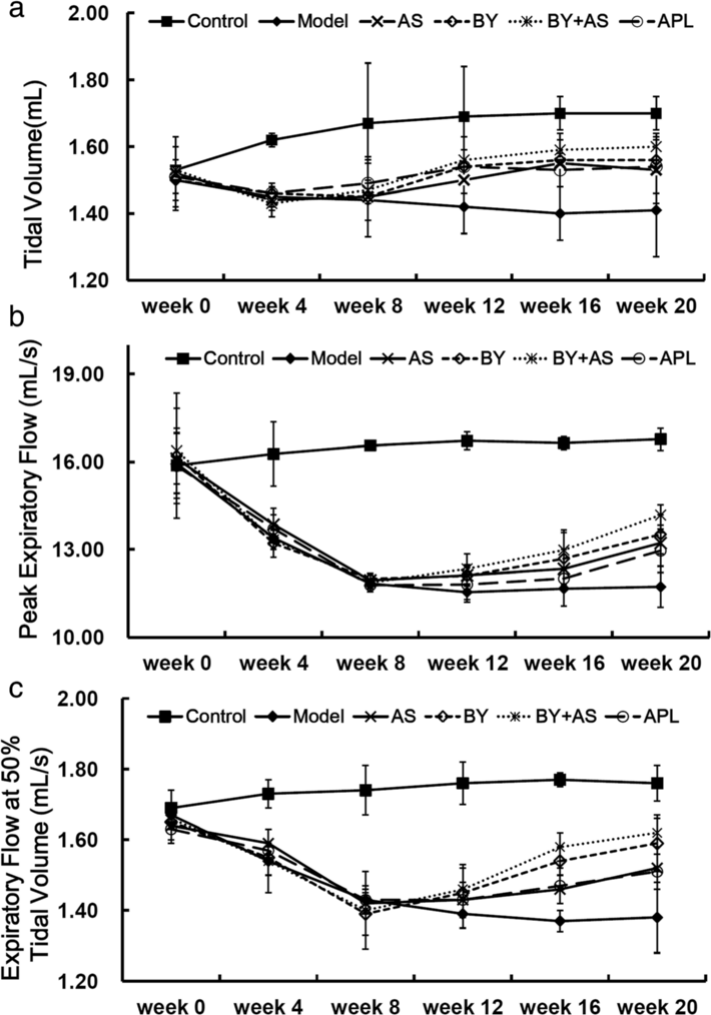
Measurement of lung function in each group at different time point. (**a**) tidal volume (V_T_), (**b**) peak expiratory flow (PEF), (**c**) peak expiratory at 50 % tidal volume (EF50). The data are expressed as mean ± SEM (*n* = 18–20). Control: control group; Model: model group; AS: acupoint sticking group; BY: Bufei Yishen group; BY + AS: Bufei Yishen + Acupoint sticking group; APL: aminophyline group

As shown in Fig. 2, from week 4, PEF and EF50 decreased in Model group compared with Control group (*P* < 0.01 or *P* < 0.05); from week 8, V_T_ in Model group was lower than Control group (*P* < 0.01). At week 16, V_T_ in BY, AS, BY + AS and APL groups was higher than Model group (*P* < 0.01 or *P* < 0.05); EF50 in BY and BY + AS groups was higher than Model group (*P* < 0.01 or *P* < 0.05). At week 20, V_T_, PEF, EF50 in BY and BY + AS groups increased compared with Model group (*P* < 0.01 or *P* < 0.05), while EF50 in AS group was higher than Model group (*P* < 0.05) and V_T_ in APL group higher than Model group (*P* < 0.05).

### Pulmonary histopathological changes

**Fig. 3:**
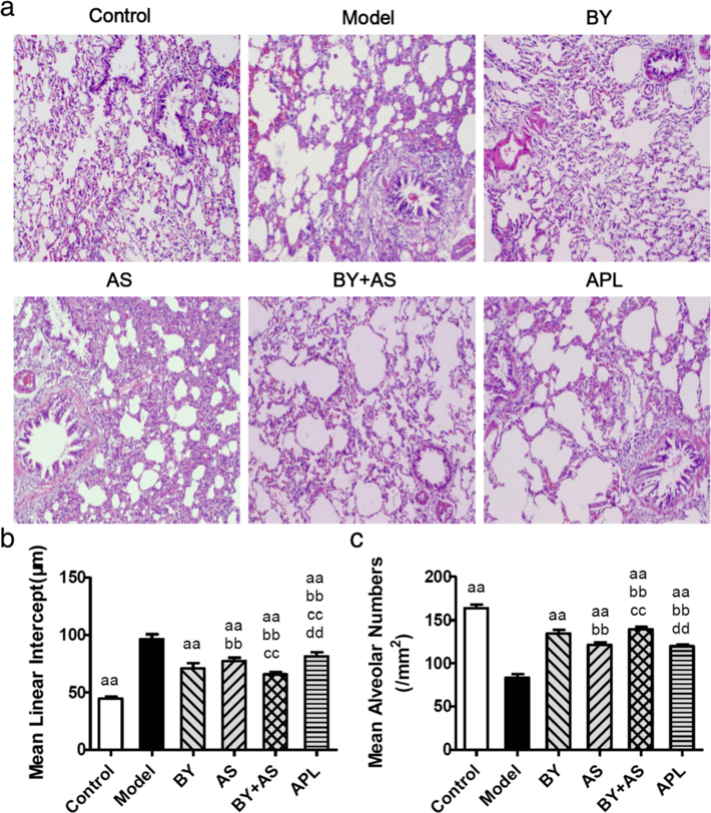
Pathological changes in the lungs of each group (H&E stained × 100) and Mean linear intercept (MLI) and mean alveolar numbers (MAN) in all groups. Control: control group; Model: model group; AS: acupoint sticking group; BY: Bufei Yishen group; BY + AS: Bufei Yishen + Acupoint sticking group; APL: aminophyline group. Values are the mean ± SEM. ^aa^ *P* < 0.01 vs Model group; ^bb^ *P* < 0.01 vs BY group; ^cc^ *P* < 0.01 vs AS group; ^dd^ *P* < 0.01 vs BY + AS group

As shown in Fig. 3a, no marked pulmonary impairment was observed in the control rats. Compared to Control group, rats in Model group showed severe pathological changes, such as bronchiole stenosis, alveolar cavity expansion, alveolar destruction, inflammatory cells infiltration and mucosal hyperplasia. The pathological changes were alleviated in the treatment groups at different degrees, especially in BY and BY + AS groups.

As shown in Fig. 3b and c, MLI increased significantly in Model group compared with Control group (*P* < 0.01), while MAN decreased significantly (*P* < 0.01). Compared to Model group, MLI decreased significantly in AS, BY, BY + AS and APL groups (*P* < 0.01), MAN increased significantly in the treated groups (*P* < 0.01). MLI was lower in BY, AS and BY + AS group than in APL groups (*P* < 0.01), and MAN was higher in BY, BY + AS groups than in APL group (*P* < 0.01). MLI was lower in BY + AS group than in BY and AS groups (*P* < 0.01), while MAN was higher (*P* < 0.01). MLI was lower in BY group than in AS groups (*P* < 0.01), while MAN was higher than in AS group (*P* < 0.01).

### The ultrastructure of respiratory membrane under electron microscopy

**Fig. 4:**
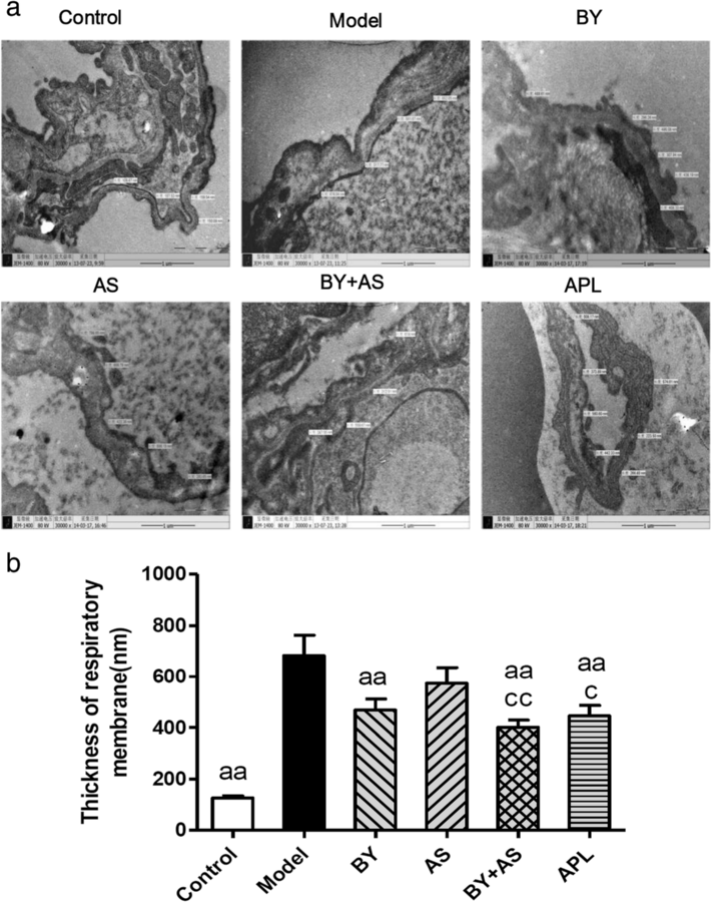
The ultrastructure of lung respiratory membrane in all groups (×30000). Control: control group; Model: model group; AS: acupoint sticking group; BY: Bufei Yishen group; BY + AS: Bufei Yishen + Acupoint sticking group; APL: aminophyline group. Values are the mean ± SEM. ^aa^ *P* < 0.01 vs Model group; ^bb^ *P* < 0.01, ^b^ *P* < 0.05 vs BY group; *n* = 8

As shown in Fig. 4a and b, the basal lamina of respiratory membrane are homogeny and the thickness was relatively homogeneous in Control group, while it in Model group was illegibility and rough, and it was significantly thicker than Control group (*P* < 0.01). The thickness of respiratory membrane in BY, BY + AS and APL groups decreased compared with Model group (*P* < 0.01), while it in BY + AS and APL groups decreased compared with AS group (*P* < 0.01 or *P* < 0.05).

### The ultrastructure of Type II alveolar epithelial cells under Electron microscopy

**Fig. 5:**
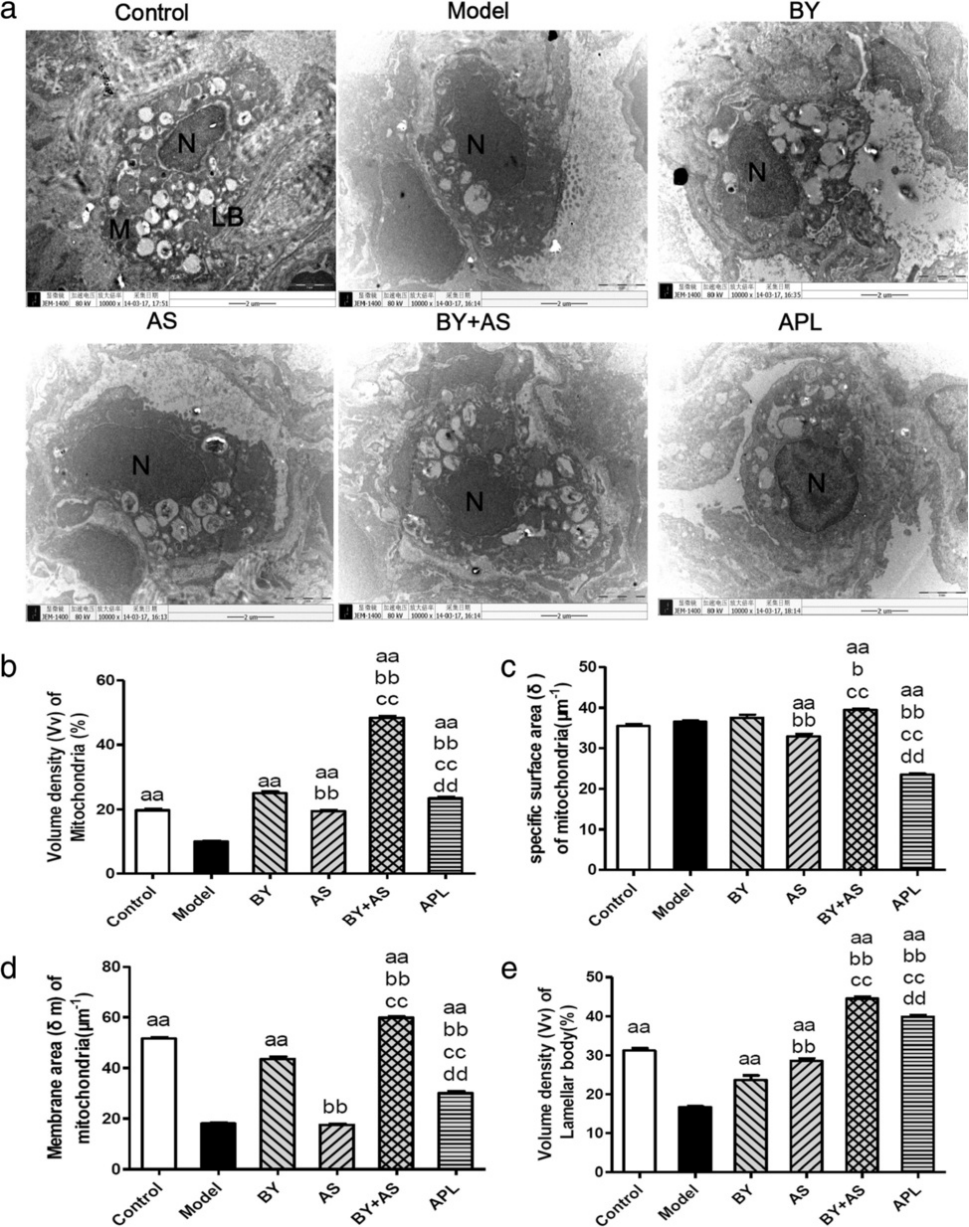
The ultrastructure of Type II alveolar epithelial cells in all groups (×10000). Control: control group; Model: model group; AS: acupoint sticking group; BY: Bufei Yishen group; BY + AS: Bufei Yishen + Acupoint sticking group; APL: aminophyline group. M: mitochondrion; N: nucleus; LB: lamellar bodies. Bar = 2 μm in (**a**, **b**, **c**, **d** and **e** represented volume density (Vv), specific surface area (δ) and ratio of membrane area (δm) of mitochondria and Vv of lamellar body in Type II alveolar epithelial cells respectively. Values are the mean ± SE. ^aa^ *P* < 0.01 vs Model group; ^bb^ *P* < 0.01 vs BY group; ^cc^ *P* < 0.01, ^c^ *P* < 0.05 vs AS group; ^dd^ *P* < 0.01 vs BY + AS group

As shown in Fig. 5a, under the electron microscope, it was observed in Control group that the pulmonary alveoli were integrated; the nuclear membrane was complete; the nuclear chromatin was uniform; and mitochondria, rough endoplasmic reticula and lamellar bodies arranged in concentric circles or in parallel were present in the cytoplasm of type II alveoli epithelial cells. In Model group, the swollen mitochondria, shortened cell ridges and the thinned microvilli were observed in type II alveoli epithelial cells, and the alveolar wall was thickened. In AS, BY and APL group, most of type II alveoli epithelial cells had shed and detached from the basement membrane, the lamellar bodies were reduced, and the microvilli were thinned and increased in number. In BY + AS group, several type II alveoli epithelial cells had detached from the basement membrane.

As shown in Fig. 5b, c, d and e, Vv and δm of mitochondrion in Model group decreased significantly compared with that in Control group (*P* < 0.01), while that in BY, BY + AS and APL groups increased significantly compared with that in Model group (*P* < 0.01), and Vv of mitochondrion in AS group was higher than that in Model group (*P* < 0.01). Vv and δm of mitochondrion were higher than in BY, BY + AS groups than in APL group (*P* < 0.01), and in AS group was lower than in APL groups (*P* < 0.01). Vv and δm of mitochondrion were higher than in BY, BY + AS groups than in AS group (*P* < 0.01).

Vv of lamellar body in Model group decreased significantly compared with in Control group (*P* < 0.01), while that in AS, BY, BY + AS and APL groups were significantly higher than in Model group (*P* < 0.01). Vv of lamellar body in BY, BY + AS and APL groups were significantly lower than in AS group (*P* < 0.01), and that in BY + AS group was significantly higher than that in AS, BY and APL groups (*P* < 0.01).

## Discussion

This study was to explore the beneficial effects of Bufei Yishen granule combined with acupoint sticking therapy on pulmonary function and morphormetry in a COPD rat model induced by cigarette-smoke and bacterial infections exposures. In this study, we found that Bufei Yishen granule and acupoint sticking can improve pulmonary function and lung pathological impairment in COPD rats, the curative effect of the combination is better than acupoint sticking or aminophylline only.

In recent years, much attention has been paid to curative effect of traditional Chinese medicine (TCM), which not only refers to internal treatment, but also external therapy due to its practical convenience and fewer side effects. In routine medication, combined internal-external therapy can improve the therapeutic effect, while there is limited evidence concerning TCM comprehensive interventions for the patients with stable COPD. Based on this, a multicentre clinical study have been carried out to confirm the efficacy of Bufei Yishen granule combined with acupoint sticking therapy in patients with stable COPD in our earlier study [7, 8]. In our present study, COPD rats were replicated and treated with Bufei Yishen granule, acupoint sticking and the combination, then lung function, lung pathology and ultrastructure were observed.

COPD belongs to the category of lung distention (Feizhang) in TCM. The pattern of lung-kidney qi deficiency is one of the most common patterns in the stable phase [13, 14]. Great curative effect has been achieved by lung-supplementing kidney-strengthening method in treating with COPD patients. In previous study, Bufei Yishen granule had good curative effect in clinic [15], as well as in animal study, such as improving immune function and alleviating inflammation [16]. Acupoint sticking therapy, a treatment which externally applying herbal paste to acupoints, is used for many lung conditions in TCM practice to nourish and warm Yang qi, remove pathogenic cold. Shu-Fei Tie acupoint sticking therapy in this study is based on TCM theory and the paste is made by pungent and warm herbs. Shu-Fei Tie acupoint sticking therapy can significantly alleviate patients’ symptoms (cough, sputum, chest distress) and quality of life [6].

Chronic airway limitation is a key pathogenesis in the process of COPD and lung function is crucial to diagnose and assess the severity of COPD. Tidal volume (V_T_), peak expiratory flow (PEF) and expiratory flow at 50 % tidal volume (EF50), which may reflect the extent of airway limitation, can be obtained easily by using unrestrained whole body plethysmography, repeatedly. Based on our results, the level of V_T_, PEF, EF50 significantly decreased in COPD rats. Bufei Yishen granule and the combination with Bufei Yishen granule and acupoint sticking can improve these indexes, while Acupoint sticking only improved EF50 and aminophylline only improved V_T_.

The alveolar structure failure, emphysema are the main pathological characteristics of COPD. Clearly visible bronchiole stenosis, alveolar cavity expansion, alveolar destruction, inflammatory cells infiltration, emphysema could be seen in COPD rats. Bufei Yishen combined with Acupoint sticking can alleviate pathological damage obviously. MLI and MAN are indicators reflected the size of alveolar cavity and the density of alveolae. Our study showed that alveolar cavity became bigger and the density of alveolae became smaller in COPD rats. All the four treatment protocols can alleviate this pathological damage, especially the combined therapy. Respiratory membrane is the principle pathway for gas exchange. Respiratory membrane thickening can be seen in injured lung tissue, which will cause low respiratory efficiency. In this study, the thickness of respiratory membrane increased significantly in COPD rats. All the four treatment protocols can decrease the thickness of respiratory membrane, especially Bufei Yishen granule combined with Acupoint sticking.

Alveolar type II epithelial cells, the major components of maintaining structure and function of pulmonary alveoli, are primarily responsible for producing, secreting and recycling surfactant. Mitochondria in type II epithelial cells are extremely important organelles for forming ATP and providing energy, while lamellar bodies store surfactant. Mitochondria volume density (Vv), specific surface area (δ) and membrane area (δm) are the main indicators to represent the number and function of mitochondria. Vv represents the number of mitochondria. The level of δ represents degree of mitochondrial Swelling. δm is positive correlated to the metabolic activity of cells [17, 18]. In this study, our data showed that Vv and δm decreased significantly in COPD rats, which indicate the reduced number and function of mitochondria in COPD rats. Bufei Yishen granule, Bufei Yishen granule combined with Acupoint sticking and aminophyline can improve the number and function of mitochondria prominently, especially Bufei Yishen granule combined with Acupoint sticking, while single Acupoint sticking only can improve number of mitochondria.

In this study, we found that Bufei Yishen granule combined with Shu-Fei Tie has beneficial effects on COPD rats, but its mechanism is not very clear. Inflammation, oxidative stress and immune dysfunction are the main pathological mechanism of COPD [1, 19]. We conclude that the effect of Bufei Yishen granule combined with Shu-Fei Tie maybe related with them. Therefore, our further study for mechanism exploration will mainly focus on the inflammatory response, oxidative stress as well as related signaling pathway, such as mitogen-activated protein kinase (MAPK), peroxisome proliferator activated receptor-γ(PPARγ) signaling pathway, which may be involved in mechanisms of drug action.

## Conclusion

Bufei Yishen granule combined with Shu-Fei Tie therapy has good effect on COPD, which can alleviate chronic airway limitation and pulmonary pathological damage, as well as function of mitochondria in alveolar type II epithelial cells. The effect of combined internal-external therapy is better than single internal or external therapy, and the mechanism need further study.
